# Comparison of Subcutaneous and Vaginal Progesterone Used for Luteal Phase Support in Patients Undergoing Intracytoplasmic Sperm Injection Cycles

**DOI:** 10.5935/1518-0557.20200090

**Published:** 2021

**Authors:** Saghar Salehpour, Nasrin Saharkhiz, Leila Nazari, Ali Sobhaneian, Sedighe Hosseini

**Affiliations:** 1 Professor, Preventative Gynecology Research Center (PGRC), Shahid Beheshti University of Medical Sciences, Tehran, Iran; 2 Associate Professor, Preventative Gynecology Research Center (PGRC), Shahid Beheshti University of Medical Sciences, Tehran, Iran; 3 Assistant Professor, Islamic Azad University, Department of Pharmaceutical Sciences; 4 Assistant Professor, Preventative Gynecology Research Center (PGRC), Shahid Beheshti University of Medical Sciences, Tehran, Iran

**Keywords:** ICSI, clinical pregnancy, ongoing pregnancy, Cyclogest, Prolutex

## Abstract

**Objective:**

Luteal phase defect in patients undergoing assisted reproductive technology (ART) is a sign of uterine failure due to insufficient progesterone effects on the endometrium. This study aims to compare the success rate and side effects of subcutaneous progesterone and vaginal progesterone to support the luteal phase in ART cycles.

**Methods:**

In this prospective randomized study, we used the traditional intracytoplasmic sperm injection (ICSI), and we transferred one or two 4-8 cell fetuses based on the patient’s age on the third day of inoculation. We started with luteal phase support from the day of oocyte recovery and the patients randomly received either a daily dose of 25mg subcutaneous progesterone (Prolutex, IBSA Switzerland) or a 400mg dose of vaginal progesterone (Cyclogest, Actoverco, United Kingdom) every 12 hours. If blood BHCG pregnancy test was positive, support for the luteal phase continued until week 10 of gestation. The measured outcomes were the clinical, chemical and ongoing pregnancy rates as well as the rate of early abortion, patients’ acceptance, tolerance and satisfaction.

**Results:**

The results of the present study showed that there was no statistically significant difference between clinical, chemical and ongoing pregnancy rates - as well as the rate of early abortion, and patients’ satisfaction when comparing the two treatment Groups.

**Conclusions:**

it seems that the subcutaneous form of progesterone can be used in patients who are not willing to use vaginal progesterone, with similar treatment results and patient satisfaction, when compared to vaginal progesterone.

## INTRODUCTION

Luteal phase deficit in patients undergoing assisted reproductive technology (ART) indicates a failure to provide uterine cavity lining at the right time due to inadequate progesterone effects on the endometrium (Pabuccu & Akar, 2012). Since the fetus implantation is largely dependent on the endometrial lining conditions, luteal phase deficit interferes with the woman’s ability to carry on her pregnancy (Shah & Nagarajan, 2013). The underlying causes of this interference include ovary overgrowth, inhibition with GnRH agonists and the use of HCG for ultimate follicular maturation (van der Gaast *et al*., 2003). Extracting granulosa cells during oocyte recovery may also result in luteal phase deficits and a change in estrogen/progesterone ratio, and high estrogen levels, which inhibits the implantation of fetuses in animals and humans (Gidley-Baird *et al*., 1986; Forman *et al*., 1985). Therefore, luteal phase support in ART cycles is an important subject.

Progesterone as a luteal phase supplement can be used in the form of 17-alpha-hydroxyprogesterone, micronized progesterone, or dydrogesterone (Devroey *et al*., 1989). Intramuscular injection of progesterone at a dose of 50mg to 100mg per day is associated with local pain, lack of patient collaboration, and other problems such as the inability of patient to self-inject, inflammation and local reactions, as well as sterile abscess in the injection site (Tavaniotou*et al*., 2000; Levine, 2000; Check, 2009; Propst *et al*., 2001; Costabile *et al*., 2001; Lightman *et al*., 1999; Silverberg *et al*., 2012; Posaci *et al*., 2000). Oral progesterone has low bioavailability and is rapidly depleted in the liver, causing side effects such as severe sleepiness and gastrointestinal problems (Arafat *et al*., 1988).

For cultural or religious reasons, some patients are reluctant to use vaginal drugs or are afraid that the dosage will not be enough, or the sufficient dose will not be absorbed. Therefore, subcutaneous administration of drugs seems to be an appropriate alternative for these patients (Donders *et al*., 2000). Prolutex is a subcutaneous complex of progesterone and hydroxypropyl-b-cyclodextrin in water, which can produce an appropriate endometrial dilatation, with a daily dose of 25mg-50 mg (Zoppetti *et al*., 2007; de Ziegler *et al*., 2013). In addition, Prolutex bioavailability is equivalent to injectable progesterone, but its absorption is faster (Baker *et al*., 2014).

Since the use of subcutaneous progesterone for luteal phase support has not been studied in our country, we aimed to compare subcutaneous progesterone (Prolutex, IBSA Switzerland) with vaginal progesterone (Cyclogest, Actoverco, United Kingdom), in infertile couples undergoing ICSE cycles, in terms of the success rate of treatment, side effects and patient satisfaction.

## MATERIALS AND METHODS

This study was a prospective randomized clinical study. The participants were infertile patients referred to the infertility clinic of Taleghani Hospital, Tehran, Iran during the months of August to March 2017. All stages of this study were approved by our institutional ethics committee prior to the implementation of the project, and we obtained an informed consent from all the participants, who were then randomly divided into two Groups. Group A received Prolutex injections and Group B received vaginal Cyclogest suppository for luteal phase support. Women aged 20 to 40 years who were treated with ICSI fresh cycle, had normal endometrial thickness (7mm-12mm) on day of embryo transfer, and had no endometrial pathology when entered the study. Women with advanced endometriosis, pelvic duct adhesion, and history of previous ICSI failures were excluded from the present study.

Patients received subcutaneous Fostimon (75 IU/ml, IBSA Swiss) from the third day of menstruation based on their age and ovarian reserve. After the size of the follicle reached 12mm-14mm all patients received GnRH antagonist protocol (subcutaneous 250 IU/ml, Cetrotide, Germany). We adjusted the Fostimon dose according to the size and number of observed follicles in the ultrasound and when two or more follicles reached 17mm to 20mm in diameter, intramuscular injection of 10000 units of HCG (IBSA, Switzerland) was performed. We recovered the oocytes 34-36 hours later.

We performed the traditional ICSI and the transfer of one or two 4-8 cell fetuses based on the age of the patient was performed on the third day of inoculation. Luteal support began on the day of oocyte recovery, and the patients randomly received either a daily dose of 25mg subcutaneous progesterone ( Prolutex, IBSA, Switzerland) or 400mg of vaginal progesterone suppository (Cyclogest, Actoverco, United Kingdom) every 12 hours. The pregnancy test using blood BHCG was performed two weeks after the embryo transfer, and if the test was positive, support for the luteal phase continued until week 10 of gestation. If the pregnancy test was positive, vaginal sonography was performed 3-4 weeks later to confirm a pregnancy sac and a clinical pregnancy. We recorded the clinical, chemical, and ongoing pregnancy rates as well as premature abortions, patients’ acceptance, tolerance and satisfaction.

### Statistical Analysis

We ran the data analysis by SPSS version 21 (Armonk, NY: IBM Corp.) using the Chi-squared, the Mann-Whitney and the Fisher exact tests. *P* values lower than 0.05 were considered statistically significant.

## RESULTS

Our study had 100 patients in each Group. Three patients in Group A were excluded due to drug reaction, and this Group was followed up with 97 patients. In Group B all patients were followed. Table 1 compares the demographic characteristics between the two Groups of patients.

**Table 1:** Patients' characteristics in the two Groups receiving Prolutex or Cyclogest

Variable	Level	Total	Treatment		*p*
			Prolutex	Cyclogest	
Age	20-25 25.1-30 30.1-35 35.1-40	24 (12.2%) 50 (25.4%) 53 (26.9%) 70 (35.5%)	13 (13.4%) 32 (33.0%) 17 (17.5%) 35 (36.1%)	11 (11.0%) 18 (18.0%) 36 (36.0%) 35 (35.0%)	0.238‡
BMI	16-20 20.1-25 25.1-30 >30.1	13 (6.6%) 108 (54.8%) 57 (28.9%) 19 (9.6%)	9 (9.3%) 63 (64.9%) 21 (21.6%) 4 (4.1%)	4 (4.0%) 45 (45.0%) 36 (36.0%) 15 (15.0%)	<0.001‡
AFC			11.87±5.97	11.77±6.82	0.912
Infertility (Type)	Primary Secondary	146 (74.1%) 51 (25.9%)	79 (81.4%) 18 (18.6%)	67 (67.0%) 33 (33.0%)	0.021*
Infertility (Cause)	Male factor PCOS Age factor Uterine factor Tubual factor Mixed Other	60 (30.5%) 10 (5.1%) 9 (4.6%) 3 (1.5%) 21 (10.7%) 66 (33.5%) 28 (14.2%)	29 (29.9%) 5 (5.2%) 3 (3.1%) 3 (3.1%) 9 (9.3%) 30 (30.9%) 18 (18.6%)	31 (31.0%) 5 (5.0%) 6 (6.0%) 0 (0.0%) 12 (12.0%) 36 (36.0%) 10 (10.0%)	0.327**
Chemical Pregnancy	No chemical pregnancy Chemical pregnancy	115 (58.4%) 82 (41.6%)	57 (58.8%) 40 (41.2%)	58 (58.0%) 42 (42.0%)	0.914*

‡ Based on Mann-Whitney test.

* Based on Chi-Square test.

** Based on Fisher exact test.

According to the data from table 1, there was no statistically significant difference between the two Groups regarding the patients age (*p*=0.238). The patients were homogeneous in terms of the cause of their infertility (*p*=0.327) and chemical pregnancy (0.914).

Table 2 depicts the results related to the main outcomes in the two treatment Groups. Clinical pregnancy in Group A was 37.1%, and in Group B it was 36%, with no statistically significant difference between the two Groups (*p*=0.871). The ongoing pregnancy in Group A was 37.1% and in Group B it was 36% (*p*=0.871). The percentage of abortions in Group A was 4.1% and in Group B it was 4% (*p*>0.99).

**Table 2:** Results related to main outcomes in the two treatment Groups

Variable	Level	Total	Treatment		Diff	95% CI		*p*
			Prolutex	Cyclogest		Lower	Upper	
Clinical Pregnancy	No clinical pregnancy	125 (63.5%)	61 (62.9%)	64 (64.0%)	1.1%	-12.5%	14.7%	0.871
	Clinical pregnancy	72 (36.5%)	36 (37.1%)	36 (36.0%)				
Ongoing Pregnancy	No ongoing pregnancy Ongoing pregnancy	125 (63.5%) 72(36.5%)	61 (62.9%)	64 (64.0%)	1.1%	-12.5%	14.7%	0.871
			36 (37.1%)	36 (36.0%)				
Abortion	No abortion Abortion	189 (95.9%) 8 (4.1%)	93 (95.9%) 4 (4.1%)	96 (96.0%) 4 (4.0%)	0.1%	-5.5%	5.7%	>0.99

Also based on our results, there was no significant relationship concerning patient satisfaction (0.549) with the type of treatment. Overall, 3.6% of patients in Group A had injection site pain and 5.2% had a brief cutaneous hypersensitivity in the injection site, while 3% of patients in Group B complained of vaginal discharge (Table 3).

**Table 3:** Comparison of patients' satisfaction and side effects between the two Groups

Variable	Level	Total	Treatment		Diff	95% CI		*p*
			Prolutex	Cyclogest		Lower	Upper	
Satisfaction	1 2	193 (98.5%) 3 (1.5%)	95 (97.9%) 2 (2.1%)	98 (99.0%) 1 (1.0%)	1.1%	-2.4%	4.5%	0.549
Discharge	No discharge Discharge	194 (98.5%) 3 (1.5%)	97 (100.0%) 0 (0.0%)	97 (97.0%) 3 (3.0%)	-3.0%	-6.4%	0.4%	0.246
Injection site Pain	No pain Yes	190 (96.4%) 7 (3.6%)	90 (92.8%) 7 (7.2%)	100 (100.0%) 0 (0.0%)	7.2%	2.1%	12.3%	0.006
Skin Hypersensitivity	No Yes	192 (97.5%) 5 (2.5%)	92 (94.8%) 5 (5.2%)	100 (100.0%) 0 (0.0%)	5.2%	0.8%	9.5%	0.027

## DISCUSSION

In the present study, we compared subcutaneous progesterone (Prolutex, IBSA Switzerland) with vaginal progesterone (Cyclogest, Actoverco, United Kingdom), for supporting the luteal phase in infertile couples undergoing ICSE cycles. We found no statistically significant difference between the two Groups of patients regarding the outcomes, including the rate of clinical pregnancy, ongoing pregnancy and abortion rate.

Similar to our findings, Baker *et al*. (2014) ran a study with 800 women undergoing IVF in 8 centers in the USA, from 2009 through 2011. The ongoing pregnancy rate per retrieval for subcutaneous (Prolutex) versus vaginal progesterone (Endometrin) was 41.6 versus 44.4%, respectively, showing non-inferiority of subcutaneous progesterone for luteal phase support. In addition, rates of initial positive β-hCG, clinical intrauterine pregnancy with fetal cardiac activity, implantation, live birth and take-home baby were comparable (Baker *et al*., 2014). Another study by Lockwood *et al*. (2014), involving 13 European fertility clinics in 2014 compared the efficacy and tolerability of Prolutex with vaginal progesterone gel (8% Crinone) in supporting the luteal phase in ATR patients). Ongoing pregnancy rates upon 10 weeks of treatment were 27.4% and 30.5% in the Prolutex and Crinone Groups, respectively, indicating non-inferiority of Prolutex compared to Crinone (Lockwood*et al*., 2014). Delivery and live birth rates were also equivalent between the two treatment Groups (Lockwood *et al*., 2014).

In a study by Doblinger *et al*. (2016), subcutaneous progesterone 25mg daily (714 patients) was compared to either progesterone vaginal gel 90 mg daily or 100 mg intravaginal twice-a-day (721 patients) for luteal phase support in IVF patients. The authors reported that the administration of progesterone versus intra-vaginal progesterone had no impact on the rate of ongoing pregnancies, live birth rate or OHSS risk.

Based on our findings, there was no significant relationship regarding patient satisfaction with the type of treatment. Overall, 3.6% of patients in the Prolutex Group had injection-site pain and 5.2% had a brief cutaneous hypersensitivity in the injection site, while 3% of patients in the Cyclogest Group complained of vaginal discharge. Similarly, in the study by Baker *et al*. (2014) comparing Prolutex versus Endometrin, both formulations were well-tolerated, with no difference in serious adverse events. Moreover, there were no statistically significant differences concerning comfort of usage and overall satisfaction between Prolutex and 8% Crinone (Lockwood *et al*., 2014).

In our study, about 25 patients in the prolutex Group and 51 patients in the cyclogest Group had BMI over 25, and overweight and obese patients may require higher initial doses of progesterone supplementation (Brady *et al*., 2014), but we used similar doses for these patients. One shortcoming of the present study is the relatively low sample size, which might affect our findings.

## CONCLUSION

The subcutaneous form of progesterone can be used with patients who are not willing to use vaginal progesterone with similar treatment results, and similar patient satisfaction when compared to vaginal progesterone.
